# Accuracy of SIAscopy for pigmented skin lesions encountered in primary care: development and validation of a new diagnostic algorithm

**DOI:** 10.1186/1471-5945-10-9

**Published:** 2010-09-25

**Authors:** Jon D Emery, Judith Hunter, Per N Hall, Anthony J Watson, Marc Moncrieff, Fiona M Walter

**Affiliations:** 1 General Practice, School of Primary, Aboriginal and Rural Health Care, University of Western Australia, 328 Stirling Highway, Claremont, WA 6010, Australia; 2 Dept of Plastic Surgery, Addenbrooke's Hospital NHS Foundation Trust, Hills Road Cambridge, CB2 2QQ, UK; 3 Welsh Centre for Burns and Plastic Surgery, Morriston Hospital, Swansea, SA6 6NL, UK; 4 Dept of Plastic & Reconstructive Surgery, Norfolk & Norwich University Hospitals NHS Foundation Trust, Norwich, Norfolk NR4, UK; 5 General Practice & Primary Care Research Unit, Department of Public Health & Primary Care, Institute of Public Health. University of Cambridge, Cambridge CB2 0SR, UK

## Abstract

**Background:**

Diagnosing pigmented skin lesions in general practice is challenging. SIAscopy has been shown to increase diagnostic accuracy for melanoma in referred populations. We aimed to develop and validate a scoring system for SIAscopic diagnosis of pigmented lesions in primary care.

**Methods:**

This study was conducted in two consecutive settings in the UK and Australia, and occurred in three stages: 1) Development of the primary care scoring algorithm (PCSA) on a sub-set of lesions from the UK sample; 2) Validation of the PCSA on a different sub-set of lesions from the same UK sample; 3) Validation of the PCSA on a new set of lesions from an Australian primary care population. Patients presenting with a pigmented lesion were recruited from 6 general practices in the UK and 2 primary care skin cancer clinics in Australia. The following data were obtained for each lesion: clinical history; SIAscan; digital photograph; and digital dermoscopy. SIAscans were interpreted by an expert and validated against histopathology where possible, or expert clinical review of all available data for each lesion.

**Results:**

A total of 858 patients with 1,211 lesions were recruited. Most lesions were benign naevi (64.8%) or seborrhoeic keratoses (22.1%); 1.2% were melanoma. The original SIAscopic diagnostic algorithm did not perform well because of the higher prevalence of seborrhoeic keratoses and haemangiomas seen in primary care. A primary care scoring algorithm (PCSA) was developed to account for this. In the UK sample the PCSA had the following characteristics for the diagnosis of 'suspicious': sensitivity 0.50 (0.18-0.81); specificity 0.84 (0.78-0.88); PPV 0.09 (0.03-0.22); NPV 0.98 (0.95-0.99). In the Australian sample the PCSA had the following characteristics for the diagnosis of 'suspicious': sensitivity 0.44 (0.32-0.58); specificity 0.95 (0.93-0.97); PPV 0.52 (0.38-0.66); NPV 0.95 (0.92-0.96). In an analysis of lesions for which histological diagnosis was available (n = 111), the PCSA had a significantly greater Area Under the Curve than the 7-point checklist for the diagnosis of melanoma (0.83; 95% CI 0.71-0.95 versus 0.61; 95% CI 0.44-0.78; p = 0.02 for difference).

**Conclusions:**

The PCSA could have a useful role in improving primary care management of pigmented skin lesions. Further work is needed to develop and validate the PCSA in other primary care populations and to evaluate the cost-effectiveness of GP management of pigmented lesions using SIAscopy.

## Background

Pigmented skin lesions are a common presenting problem in general practice and, while the majority are benign naevi or non-melanocytic lesions (seborrhoeic keratoses, haemangiomas), a small minority are malignant melanomas. Melanoma is a serious skin cancer, responsible for 2% of all cancers and 1% of all cancer deaths in the UK, with about 8,000 new cases and 1,800 deaths a year [1]. Worldwide, the incidence of melanoma is increasing faster than any other solid cancer with an approximate doubling of rates every 10-20 years in countries with Caucasian populations [2,3].

Pigmented lesions and melanoma pose particular diagnostic and management challenges for general practitioners (GPs) [4]. GPs are less able than dermatologists to differentiate melanomas from other pigmented lesions [5,6], probably because an individual GP will encounter melanoma infrequently [7]. British data following the establishment of urgent referral pathways for all suspected skin cancers [8] showed that only 12% of referred lesions were diagnosed as skin cancer and only 42% of skin cancers were referred via this route [9]. There have been conflicting findings about the performance of GPs who have been trained in melanoma diagnosis either face-to-face [10] or via the internet [11]. In a primary care setting the ability to distinguish benign from suspicious lesions is as important as a clinical diagnosis of melanoma in making the decision either to reassure or to refer urgently for dermatological review. Studies of diagnostic accuracy and decision aids for use in primary care need to reflect the diagnostic distinction between suspicious and benign lesions as well as the identification of melanomas.

New approaches are required to improve GPs' assessment of pigmented skin lesions. Dermoscopy has been shown to improve the diagnostic accuracy for melanoma in the specialist setting [12] and in two randomised controlled trials in general practice [13,14]. However, dermoscopy is a relatively time-consuming technique to learn; in a recent trial of dermoscopy and digital monitoring Australian GPs required up to 30 hours of internet-based learning to acquire adequate skills and only 63% of those trained actually recruited patients into the trial. There is also current interest in teledermatology but, for suspicious pigmented lesions, it is unlikely to dramatically reduce the need for conventional clinical consultations with experts whilst maintaining clinical safety [15].

**Figure 1:**
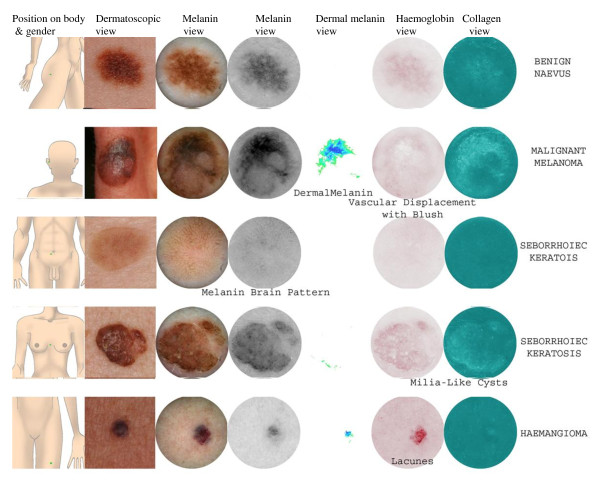
**Example SIAscans, with labelled features, of a) benign naevus b) malignant melanoma c) seborrhoeic keratosis d) haemangioma**.

An innovative approach uses SIAscopy, a non-invasive multispectral scanning technique which gains micro-architectural information about the skin within seconds. The device shines near infrared and visible spectra light from a handset through the skin. The light remitted can then be calibrated for papillary dermis thickness, using information from the infrared wavebands. The amount of dermal blood is obtained by de-referencing a given colour location on the surface of normal skin colouration. If melanin is present in the dermis, its presence can be detected from the fact that even after the papillary dermis thickness adjustment, the colours still do not lie on the surface of normal skin colouration. The amount of epidermal melanin is obtained by de-referencing skin colour locations on the surface of normal skin colouration. Within seconds all of this information is displayed graphically on the computer screen as SIAscans. SIAscans are therefore high-resolution images of the collagen and haemoglobin content of the papillary dermis, and melanin content of the epidermis and papillary dermis. Patterns within the SIAscans of pigmented skin lesions (such as the presence of dermal melanin and blood displacement with erythematous blush) indicate the pathological changes consistent with melanoma. Previous studies have demonstrated the diagnostic accuracy of SIAscopy for melanoma amongst patients referred to secondary care using the Moncrieff scoring system [16,17]. In that study the combination of the following features was found to be sensitive and specific for the diagnosis of melanoma: presence of dermal melanin, collagen holes, erythematous blush and blood displacement (see Figure 1). However, the findings of diagnostic studies on referred populations cannot be applied to patients seen in primary care due to the potential for spectrum bias. The primary aim of this study therefore was to develop and validate a scoring system for SIAscopic diagnosis of pigmented skin lesions encountered in primary care. In addition, since all studies to date on SIAscopy have been conducted in the UK, we aimed to validate the technique in an Australian primary care setting to examine its generalisability to populations with greater sun-related skin damage.

## Methods

This study was conducted in two consecutive settings in the UK and Australia and entailed the following three stages:

1. Development of the primary care scoring algorithm (PCSA) on a sub-set of lesions from the UK sample (UK Development lesion dataset);

2. Validation of the PCSA on a different sub-set of lesions from the same UK sample (UK Validation lesion dataset);

3. Validation of the PCSA on a new set of lesions from an Australian primary care population (Australian Validation lesion dataset).

### Ethical approval

Ethical approval for the UK component of the study was obtained from the Cambridge Research Ethics Committee (REC Ref. 04/079) and research governance approval from Cambridge City and South Cambridgeshire Primary Care Trusts (Project number L00569). Ethical approval for the Australian component of the study was obtained from the University of Western Australia's Human Research Ethics Committee (HREC Ref. RA/4/1/1739).

### Settings

#### UK setting

Six general practices were recruited from Cambridge city and the surrounding suburban and rural areas covering a registered population of 52,913. Adult patients aged over 18 years were recruited into the study by their general practitioner (GP) if they had presented with concerns about a pigmented skin lesion: as these were lesions presented by the patients to their GP, they included lesions that were ultimately considered not clinically suspicious. Participants were formally consented and data collected about their lesion by JH within two weeks of initial presentation to their GP. Data collection occurred between January 2005 and January 2006.

#### Australian setting

Three primary care skin cancer clinics operated by GPs were recruited from the metropolitan area of Perth, Western Australia. Adult patients aged over 18 years were recruited into the study by their GP if they presented with concerns about a pigmented skin lesion: again, these included lesions that ultimately were considered not clinically suspicious. Additional lesions were also included when a pigmented skin lesion was identified as potentially suspicious during their clinical examination. Participants were formally consented and data collected by AJW on the same day as they presented to their GP. Data collection occurred between April 2008 and January 2009.

### Data collection

The following data were collected by the medically qualified researchers (JH or AJW) for each skin lesion:

1. 7-point melanoma checklist^11^;

2. Macroscopic digital photograph (Canon EOS 400 D camera, Canon EF-S60 macro lens, Canon MR-14EX Macro Ring Lite flash, JPEG picture format: 3888 × 2592 pixels);

3. Dermoscopic digital photograph (Canon EOS 400 D camera, Canon EF-S60 macro lens, Heine SLR Photadaptor, Heine Delta 20 dermatoscope, JPEG picture format: 3888 × 2592 pixels);

4. SIAscan (MoleMate™SIAscope V and Microsoft Windows™application).

### SIAscan assessment

SIAscan images and data (including the location of the lesion and the age group and sex of the patient) were assessed by a SIAscopy expert, who was blinded to the 7-point melanoma checklist results and clinical photographs. The SIAscopy expert scored the presence or absence of each specific SIAscopic feature including those previously associated with melanoma [16]: size of lesion, age of patient, dermal melanin, collagen holes and blood displacement with erythematous blush. Additional features that were also scored were: blood vessels, white dots on the collagen view, blood lacunes and a cerebriform melanin pattern (see Figure 1).

### Diagnostic reference standards

Given that it would have been ethically unacceptable to obtain histological diagnosis on every recruited lesion, we applied the following hierarchical approach to reference standard diagnosis:

1. Histopathology.

2. In-person clinical review of the lesion by one expert, including 7-point checklist and digital dermoscopy.

3. Clinical diagnosis made on the basis of the 7-point checklist, photographic and dermoscopy images.

The expert reviewers were blinded to the SIAscan images. For the reference standard diagnosis we categorised lesions in two complementary ways relevant to primary care decision-making: (1) 'suspicious' or benign and (2) melanoma or other pigmented lesion. The definition of 'suspicious' was a lesion that, if seen in general practice, would warrant referral, excision or short-term monitoring.

### Analysis

This was undertaken in three stages:

(a) ***Development stage: ***a 66% sub-sample of lesions (UK Development lesion dataset) was scored using the Moncrieff scoring system. In order to account for the different prevalence of certain pigmented skin lesions seen in primary care, a Primary Care Scoring Algorithm (PCSA) was developed.

(b) ***Validation stage one: ***the PCSA was validated against the remaining 33% sub-sample of lesions (UK Validation lesion dataset).

(c) ***Validation stage two: ***the PCSA was validated against the lesions recruited in Australia (Australian Validation lesion dataset).

Data were recorded on a Microsoft Access database and analysed with Microsoft Excel and SPSS version 11.5 for Windows. Sensitivity, specificity, positive and negative predictive values and their associated 95% confidence intervals were calculated using standard approaches including the Wilson method to account for small sample sizes in some of the cells [18]. Receiver operating characteristic (ROC) curves and associated area-under-the-curve (AUC) were created using standard functions within SPSS 11.5 to explore different cut-off scores for the PCSA. We compared the area under the curve for the PCSA and the 7-point checklist for all lesions for which we had obtained histology, using the manual method described by Hanley and McNeil [19].

## Results

In the UK dataset (development and validation lesions) interpretable images were obtained on 630 lesions from 389 patients. The mean age of participants in the study was 44.9 years; 68.6% were female. In the Australian dataset (validation lesions) interpretable images were obtained on 581 lesions from 469 patients. Fifty two per cent of the subjects were male, and the mean age of participants was 50 years. Table 1 shows the types of lesion represented in the two datasets based on histopathology where known, or expert clinical diagnosis.

**Table 1:** Distribution of lesions in Development and Validation datasets, based on expert clinical diagnosis or histology where available

Diagnosis	Development dataset	%	UK validation dataset	%	Australian validation dataset	%
Naevus	293	69. 4%	159	76. 4%	333	57. 3%

Seborrhoeic keratosis	101	23. 9%	39	18. 7%	128	22. 0%

Solar lentigo	0	0	0	0	67	11. 5%

Basal cell carcinoma	0	0	0	0	22	3. 8%

Melanoma	3	0.7%	2	1. 0%	7	1. 2%

Angiokeratoma	0	0.0%	0	0.0%	6	1. 0%

Dermatofibroma	14	3. 3%	6	2. 9%	5	0.9%

Lentigo maligna	0	0.0%	0	0.0%	4	0.7%

Haemangioma	11	2. 6%	2	1. 0%	0	0.0%

Lentigo simplex	0	0.0%	0	0.0%	5	0.9%

Ephilis	0	0.0%	0	0.0%	3	0.3%

Papilloma	0	0.0%	0	0.0%	1	0.2%

**Total**	422		208		581	

### (a) Development stage

Table 2 presents the performance of the Moncrieff scoring system for the diagnosis of 'suspicious' using the Development Lesion dataset (n = 422). In this subset there were 24 suspicious lesions and 3 melanomas, including 1 atypical melanoma with significant regression. The Moncrieff scoring system did not perform that well for the diagnosis of 'suspicious', predominantly due to misclassification of seborrhoeic keratoses and haemangiomas. In particular, of the 101 seborrhoeic keratoses in the sample, 55 were misclassified as suspicious due to apparent 'dermal melanin' on the SIAscopic image. Specific SIAscopic features of seborrhoeic keratoses were identified as: white dots on the collagen view, analogous to milia-like cysts seen on dermoscopy [20], and a cerebriform appearance on the total melanin view. Haemangiomas were identified by the presence of blood lacunes on the SIAscan 'blood' view.

**Table 2:** Performance characteristics of SIAscopy for the diagnosis of 'suspicious' in the different datasets of lesions.

	Development dataset (Moncrieff scoring system)	UK validation dataset (PCSA)	Australian validation dataset (PCSA)
**Sensitivity** **(95% CI)**	0.54 (0.35 - 0.72)	0.50 (0.18-0.81)	0.44 (0.32-0.58)

**Specificity** **(95% CI)**	0.77 (0.73 - 0.81)	0.84 (0.78-0.88)	0.95 (0.93-0.97)

**Positive predictive value** **(95% CI)**	0.12 (0.075 - 0.20)	0.09 (0.03-0.22)	0.52 (0.38-0.66)

**Negative predictive value** **(95% CI)**	0.96 (0.93 - 0.98)	0.98 (0.95-0.99)	0.95 (0.92-0.96)

**Figure 2:**
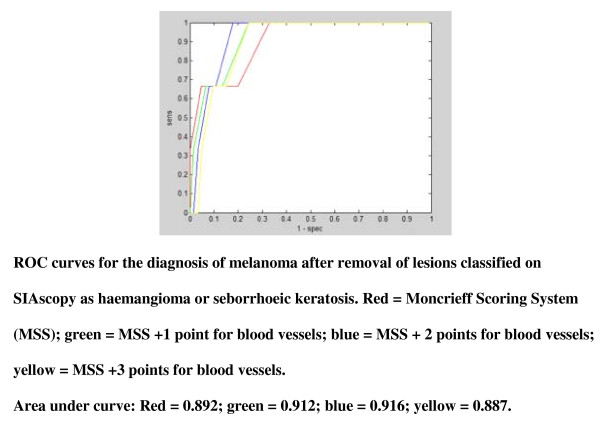
**ROC curves to show development of Primary Care Scoring Algorithm; ROC for diagnosis of melanoma after removal of lesions classified on SIAscopy as haemangioma or seborrhoeic keratosis**.

**Figure 3:**
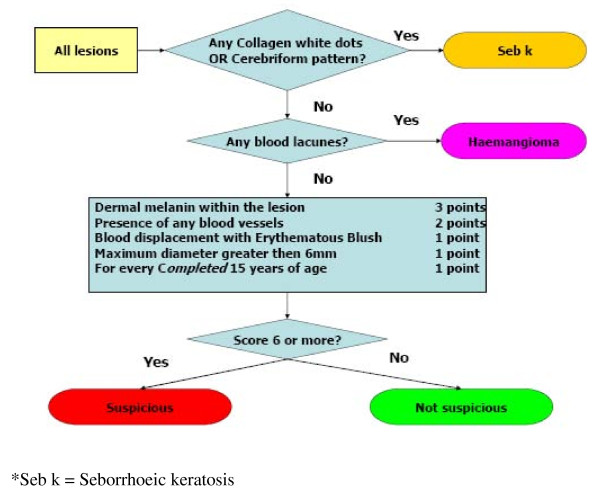
**Primary Care Scoring Algorithm (PCSA)**.

Because of this potential to misclassify seborrhoeic keratoses and haemangiomas, we therefore set out to develop a new diagnostic algorithm to improve SIAscopy's ability to distinguish these lesions, which are more prevalent in primary care than in referred populations, from melanoma. An additional feature, the presence of blood vessels, was entered into the Moncrieff model to examine its role in improving the diagnostic performance. ROC curves were plotted, using data from the Development Lesion dataset, to examine the different point scores for the presence of blood vessels. The performance of the Moncrieff score for 'suspicious' was improved if lesions classified as seborrhoeic keratoses or haemangiomas, based on SIAscopic features, were excluded from the dataset (Area Under Curve (AUC): MSS 0.732; MSS after exclusion of seborrhoeic keratoses and haemangiomas 0.759). For the diagnosis of melanoma, performance was improved by scoring 2 points for the presence of blood vessels and by excluding lesions classified as seborrhoeic keratoses or haemangiomas, based on SIAscopic features (AUC 0.916, see Figure 2). On this basis a new Primary Care Scoring Algorithm (PCSA) was developed that aims to identify lesions with features of seborrhoeic keratoses or haemangiomas first and then apply a scoring system based on the presence of other features associated with melanoma (see Figure 3). In this way, seborrhoeic keratoses and haemangiomas are no longer misclassified.

### (b) UK Validation stage

The new PCSA was tested against the 208 lesions in the Validation Lesion dataset, which included 6 suspicious lesions and two histopathologically confirmed melanomas. The performance of the PCSA is presented in Table 2.

### (c) Australian Validation stage

The PCSA was tested against the 581 lesions recruited in Australia. There were 52 suspicious lesions including 5 histopathologically confirmed melanomas and 2 lentigo malignas. The performance of the PCSA is presented in Table 2. In this second validation stage, the sensitivity for the diagnosis of suspicious was similar to the UK findings (0.44; 95% CI 0.32-0.58). However, specificity was significantly better (0.95; 95% CI 0.93-0.97). Furthermore, due to the higher prevalence of suspicious lesions in the Australian dataset, the positive predictive value for the diagnosis of suspicious was 0.52 (95% CI 0.38-0.66) while maintaining an acceptably high negative predictive value (0.95; 95% CI 0.92-0.96).

**Figure 4:**
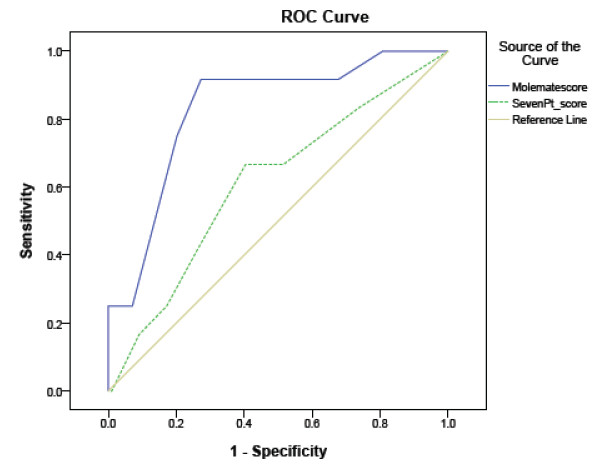
**Comparative ROC curves for the Primary Care Scoring Algorithm and 7-point checklist for lesions with a histological diagnosis (n = 111; 10 melanoma and 2 lentigo maligna)**.

We compared the AUC for the PCSA and the 7-point checklist for the 111 lesions for which we had histological diagnosis (n = 42 UK dataset; n = 69 Australian dataset; included 10 melanomas and 2 lentigo maligna) (Table 3; Figure 4). The PCSA had a significantly greater AUC (0.83; 95% CI 0.71-0.95) than the 7-point checklist (AUC 0.61; 95% CI 0.44-0.78; p = 0.02 for difference).

**Table 3:** Distribution of lesions for which histological diagnosis was available.

Diagnosis	Number	%
Naevus	62	55. 9

Seborrhoeic keratosis	16	14. 4

Melanoma*	12	10.8

Basal cell carcinoma	9	8. 1

Solar lentigo	5	4. 5

Dermatofibroma	5	4. 5

Lentigo simplex	2	1. 8

TOTAL	111	

*includes 2 lentigo maligna

## Discussion

This is the first study to test the use of SIAscopy for lesions encountered in a primary care setting and also outside the UK. We have applied a systematic approach in which we tested the initial Moncrieff scoring system to see how it would function on lesions presented in a primary care setting. This is an important step in studies of new diagnostic techniques to reduce the effects of spectrum bias. In addition to developing a new diagnostic algorithm we have conducted a second validation study on a different primary care population. This second validation study was conducted in an Australian primary care setting which, except for a higher prevalence of solar lentigos, had a similar prevalence of lesions to the UK dataset. We accept that a limitation of this study is our inability to obtain histopathological diagnoses on all the lesions recruited, but this would have been ethically unacceptable. To inform the clinical-expert reference-standard diagnosis we deliberately chose to obtain maximum clinical data, including the 7-point checklist and dermoscopy. We chose to do this so we could be as accurate as possible with our reference diagnosis where histology was not available. It is also theoretically possible that some amelanotic melanomas were not recruited into the study on the basis of our inclusion criteria. Furthermore, we did not follow-up any lesions determined as benign so it is theoretically possible that some clinically significant lesions may have been missed by our reference standard diagnoses.

The Moncrieff scoring system was found to be less accurate than in the secondary care setting due to the different prevalence of lesions among the primary care population. In order to account for the higher prevalence of non-melanocytic lesions, such as seborrhoeic keratoses and haemangiomas, we developed a new Primary Care Scoring Algorithm which was more specific than the Moncrieff scoring system for 'suspicious' but no more sensitive. Higher specificity was particularly identified in the Australian dataset suggesting that sun-related skin damage does not adversely affect the diagnostic accuracy of SIAscopy. The current algorithm accounts for size of lesion (> 6 mm) and age of patient. The mean age of participants from each studied population was 45 and 50 years respectively. It is not possible therefore to comment on the performance of the PCSA in elderly populations in which the algorithm may become less specific. It was reassuring that the PCSA's moderate sensitivity for 'suspicious' does not appear to be reflected in its sensitivity for melanoma, but inevitably there were too few melanomas in this study to provide robust estimates of diagnostic accuracy for melanoma in primary care. In subsequent research, simulation modelling of the PCSA in which a higher prevalence of melanomas was entered into the dataset suggests high sensitivity and specificity to detect melanoma [21]. Ultimately, a primary care algorithm should be good at identifying 'suspicious' pigmented lesions, including melanoma, as well as accurately ruling out lesions which are unlikely to be clinically significant. It is interesting that the PCSA appears to be more accurate than the 7-point checklist in diagnosing melanoma. The 7-point checklist was completed by the two medically qualified and trained researchers including one who was a plastic surgeon (JH). The relatively poor performance of the 7-point checklist cannot be explained by inconsistent application of the items. There were several non-melanocytic lesions which were thought clinically to be pigmented. The 7-point checklist, and Siascopy, are intended for use only with melanocytic lesions, although in clinical primary care practice this distinction can sometimes be difficult. The inclusion of non-melanocytic lesions may partially explain the poorer performance of the 7-point checklist compared to previously published data.

The analysis conducted assumes that there was no selection bias in the sampling of lesions chosen for biopsy. This is theoretically possible, for example if the 7-point score were used to inform the clinical decision to excise, and so our finding should be interpreted with some caution[22].

In this study we used experts in SIAscopy to interpret the SIAscans. This therefore reflects the best performance of SIAscopy in primary care and not how it would perform in the hands of general practitioners. As this is the first study of SIAscopy on lesions from primary care, we needed to determine the best possible performance of the technique in experienced hands. We are now conducting a randomised controlled trial of training English general practitioners in SIAscopy, including the application of the PCSA, to determine its effects on clinical practice (the MoleMate UK Trial). This trial will provide further evidence on the diagnostic accuracy of the PCSA when used by general practitioners as well as the cost-effectiveness of SIAscopy in English primary care.

We believe that the features of SIAscopy may be a great deal easier to learn than those of dermoscopy which can take a long period of training in which to become proficient. A recent study we have conducted suggests that SIAscopy features can be learnt by general practitioners using a CD-rom based tutorial in approximately two hours [23]. In a recent trial in general practice, dermoscopy had a sensitivity of 55% and specificity of 89% for malignant lesions which is comparable with our findings for SIAscopy, albeit in expert hands [14]. The MoleMate UK Trial will provide more comparable data in due course [24]. While there is no doubt that dermoscopy and digital monitoring can significantly improve the management of pigmented lesions in primary care, we believe that SIAscopy could be simpler to learn and may therefore have greater utility for a wider group of primary care practitioners than dermoscopy.

## Conclusions

The PCSA for SIAscopy could have an important role in improving the management of pigmented skin lesions in primary care. This study has confirmed the key diagnostic features of lesions commonly encountered in primary care. Further work is required to determine the impact of training GPs in SIAscopy on their clinical management of pigmented skin lesions, and the quality of their referrals to the secondary care skin cancer clinics.

## Competing interests

Part of the Australian study was funded by Astron Clinica who formerly produced devices based on SIAscopy. Astron Clinica had no input into the analyses or drafting the manuscript. From August 2009, MoleMate, SIAscopy, SIAscan and SIAscope are trademarks of Biocompatibles UK Limited, who have funded maintenance of the Australian dataset.

## Authors' contributions

JE, PH, and FW conceived the study and contributed to the overall design. JH conducted the UK Development and Validation studies as part of her MD thesis. All authors contributed to the conduct of the study and the final manuscript.

## Pre-publication history

The pre-publication history for this paper can be accessed here:

http://www.biomedcentral.com/1471-5945/10/9/prepub
